# Skin of Color Is Underrepresented on Instagram: Assessing South Jersey Skin Talk as an Educational Tool to Increase Awareness

**DOI:** 10.7759/cureus.47388

**Published:** 2023-10-20

**Authors:** Rebecca Fliorent, Sonam Mistry, Kiran Javaid, Katharine Milani

**Affiliations:** 1 Molecular Biology, Rowan-Virtua School of Osteopathic Medicine, Stratford, USA

**Keywords:** underrepresented, diversity and inclusion, skin conditions, skin tone, representation, skin of color, dermatology, social media, instagram

## Abstract

Background

Instagram, a widely used social media platform with over two billion active users, has the potential to propagate dermatologic health information within the public sphere. However, there is a lack of representation of people of color (POC), making it crucial to share accurate and inclusive posts to increase awareness about dermatologic conditions. It is also necessary to address the misconceptions about skin diseases and other hereditary conditions within various ethnic groups. To combat this, a group of medical students created South Jersey Skin Talk (SJST), an initiative aimed to improve dermatologic health literacy in skin-of-color communities, particularly in underserved areas like Camden County, NJ. Using reliable sources to prevent the spread of misinformation, SJST’s accessible Instagram page explains skin conditions, especially emphasizing appearances and manifestations in POC. The hypothesis being investigated by this group is that the implementation of SJST as a community intervention is expected to improve dermatologic health literacy in POC.

Methods

A 13-question survey was conducted via Qualtrics (Seattle, Washington) and was distributed on social media (Instagram, Facebook, Reddit, Twitter, TikTok, and GroupMe). It remained open for eight weeks during which users 18 years or older were invited to participate. The survey was divided into four sections: demographics, Instagram usage, knowledge of dermatology, and inclusion and diversity on SJST’s page. A total of 184 total responses were collected, which were compared using chi-squared analyses on Qualtrics software.

Results

POC felt less represented on social media compared to White respondents prior to visiting SJST on Instagram (p < 0.00001). However, after viewing SJST, 87.5% of White participants and 88% of POC reported feeling represented on the page. Additionally, both groups of respondents indicated that they felt more knowledgeable about their primary skin concern after viewing the SJST’s posts. Furthermore, 86.8% of POC reported that they would feel more confident participating in a conversation with their dermatologist regarding their primary dermatologic concern.

Conclusion

SJST is a community outreach organization focused on improving health literacy for POC and bridging the gap in healthcare disparities between White and POC populations. The results from this survey confirm the hypothesis and illustrate that community interventions targeted at education for POC increase health literacy and patient autonomy. These results also show that there is a need for more representation and diversity in medical dermatology on social media. Further studies should be done to investigate other disparities affecting adequate representation for POC.

## Introduction

Instagram is one of the most popular social media platforms with over two billion active monthly users and therefore has the potential to play an influential role in shaping the narrative of dermatologic health information relayed to the public [1]. With massive amounts of information accessible at one’s fingertips, it is challenging to decipher accurate dermatologic health information. Despite this vast volume of content, there is a lack of representation of people of color (POC) [2]; this creates a need for sharing both accurate and inclusive posts on social media. According to a recent study on the analysis of Instagram and skin-of-color (SOC) posts, 80% of dermatology-related posts represented lighter skin tones (Fitzpatrick types I-III), while the remaining 20% covered SOC (Fitzpatrick types IV-VI) [3]. However, the top three ethnicities using Instagram are comprised of Latino, Asian, and African American users [4]. Instagram also has the ability to correct generational biases and misconceptions related to dermatology in all ethnic groups and increase awareness of various conditions affecting these groups. For example, a study delineated that Black patients have a lower perceived risk of melanoma; however, Black individuals have an estimated 20% higher mortality rate than White individuals [5,6].

Skin conditions manifest in different ways on darker skin tones, which are often absent or lacking in major marketing campaigns. POC are most commonly affected by acne, melasma, vitiligo, and alopecia [7]. Women of color, in particular, represent a greater percentage of patients seeking dermatological care for these conditions [7]. In addition, melasma is an acquired pigment disorder that more commonly affects older women of color, characterized by excess melanin production as a result of aging and sun damage. Certain hair practices utilized among women of African descent can possibly lead to specific types of hair loss such as traction alopecia, trichorrhexis nodosa, and central centrifugal cicatricial alopecia. Lastly, while acne affects people with all skin tones, patients with darker skin tones often present with hyperpigmentation following acne, which can be difficult to manage [7].

Despite the fact that darker skin tones make up a large percentage of the American population, lighter skin tones are more represented in dermatologic educational sources [8-12]. A recent study showed that in an analysis of 4,146 textbook images from a sample of four general preclinical anatomy textbooks (i.e., Atlas of Human Anatomy, Bates’ Guide to Physical Examination and History Taking, Clinically Oriented Anatomy, and Gray’s Anatomy for Students) (2013-2015 editions), only 4.5% of images represented darker skin tones [13]. Additionally, the majority of question banks that medical students use to prepare for medical board exams only contain 20.9% of SOC images [14]. There is a similar lack of representation on social media as well. A study analyzing skin type representation in popular dermatology-related posts on Instagram found that the mean follower count for dermatology accounts with lighter skin tones was 167,660, while the mean follower count for accounts of color was 87,440 [15]. Additionally, a study analyzing hashtags on Instagram showed that while there were 1,852,029 hashtags used for acne-related searches, there were only 47,021 hashtags on hyperpigmentation, a condition more commonly experienced by people of color [16].

Many studies have suggested that the key to increasing the representation of people with SOC is increasing interventions and community outreach [6,17,18]. To help combat this, a group of medical students created an initiative called South Jersey Skin Talk (SJST). SJST is among the first interventions aimed at improving dermatologic health literacy in people with SOC in underrepresented areas, particularly in Camden County, NJ. SJST (@sjskintalk) is a public page that can be easily accessed by anyone on Instagram. SJST’s posts explain and describe different skin diseases and hereditary conditions, particularly highlighting the appearances and manifestations of SOC. Additionally, SJST aims to delineate dermatologic information in a way that anyone can understand. SJST’s information comes from reliable sources, such as the American Academy of Dermatology, dermatologic textbooks available through the Rowan-Virtua School of Osteopathic Medicine (RowanSOM) library, and peer-reviewed journals in order to prevent the spread of misinformation, which is a common issue with pages run by non-medical parties [19,20].

This study highlights the significance of enhancing education and raising awareness about skin conditions impacting POC. Furthermore, this study presents a model for utilizing inclusive social media accounts, like SJST, to expand the scope of dermatologic education available for individuals with SOC on social media platforms.

## Materials and methods

In order to assess the effectiveness of SJST in achieving its objective of enhancing diversity in dermatology on social media, a 13-question survey was distributed through Qualtrics on various social media platforms (Instagram, Facebook, Reddit, Twitter, TikTok, and GroupMe). It remained open for eight weeks during which users who were 18 years and older were invited to participate. The survey was divided into four sections: demographics, Instagram usage, knowledge of dermatology, and inclusion and diversity on SJST’s page. The demographics section consisted of the participant’s age, ethnicity, skin tone, and primary skin concerns (acne, hyperpigmentation, eczema, mole, etc.). Respondents were asked to rank their skin tone on the Monk skin tone scale, a revamped version of the Fitzpatrick scale that contains a broader spectrum of skin tones.

Information regarding participants’ social media usage for skin care and skin education was collected using a Likert scale ranking system (Table 1). The next section was essential in assessing the effectiveness of SJST as an educational tool. It included four “yes/no” questions to investigate respondents’ health literacy after looking at the SJST’s Instagram page. The next section of the survey evinced whether or not SJST helped establish patient autonomy in their dermatologic treatment plan.

**Table 1:** Survey questionnaire This survey was deployed via Qualtrics on various social media sites with information directing participants to view @SJST prior to taking the survey. Additional information on participant's eligibility and IRB approval was also provided prior to the start of the survey. SJST: South Jersey Skin Talk.

Question	Response options					
Demographics						
Age (years)	18-24	25-34	35-44	45-54	55-64	>65
Ethnicity	White	Hispanic or Latino	Black or African American	Native American or American Indian	Asian or Pacific Islander	Other
Skin tone	A through J, corresponding to Monk scale image selection					
Social media usage						
Which social media platform do you use the most?	TikTok	Instagram	Facebook	Snapchat	Reddit	Twitter
How helpful do you find Instagram as an educational tool for skincare?	1 - "Not at all"	2	3	4	5 - "Very helpful"	
How useful do you find Instagram as an educational tool for skin conditions?	1 - "Not at all"	2	3	4	5 - "Very helpful"	
Dermatologic health literacy						
How much did you know about skin conditions (e.g., acne, eczema, etc.) prior to looking through @sjskintalk’s profile?	1 - "None"	2	3	4	5 - "Very much"	
How much did you learn about skin conditions (e.g., acne, eczema, etc.) after looking at @sjskintalk’s profile?	1 - "None"	2	3	4	5 - "Very much"	
Would you feel more knowledgeable in the decision-making process for dermatologic treatments after looking at @sjskintalk’s profile?	Yes	No				
Would you feel more knowledgeable asking your dermatologist questions after viewing @sjskintalk’s page?	Yes	No				
Would you be more likely to see a dermatologist after viewing @sjskintalk’s page?	Yes	No				
Would you feel more knowledgeable seeing a dermatologist after looking at @sjskintalk’s profile?	Yes	No				
Do you feel like @sjskintalk uses easy-to-understand language to describe skin conditions?	Yes	No				
Which skin conditions are your primary concerns? (Select all that apply)	Acne	Eczema	Psoriasis	Pigmentation	Aging	Other

Feedback about the level of diversity and inclusion on the page itself compared to other pages/sources was collected in the final section. This section assessed whether or not SJST executed its goals: to represent POC in dermatology and delineate accessible information. Survey responses were collected and compared using chi-squared analysis utilizing appropriate Qualtrics software.

## Results

**Figure 1:**
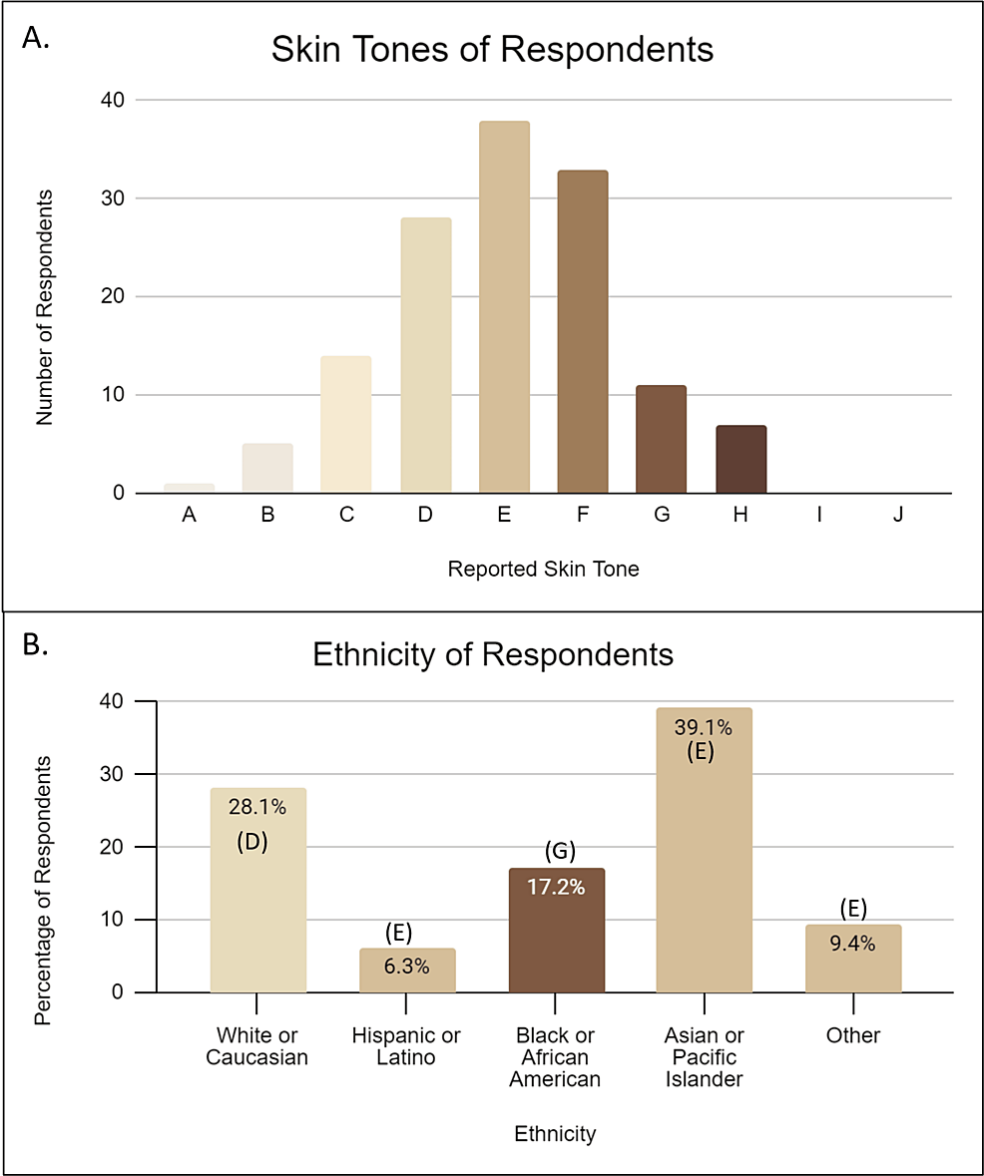
Skin tone and ethnicity of survey participants (A) This graph represents the distribution of skin tones of respondents separate from their ethnicity. Participants selected the skin tone that best reflected their own appearance using the Monk scale. (B) This graph represents the ethnicity selected by the survey respondents. The color of the bars and the letters represent the mean skin tone selected by the participants in the groups on the Monk scale (n = 184).

A total of 184 individuals took this survey. Information on the respondents' skin tone was collected using the Monk scale, and a normal distribution was observed (Figure 1, Panel A). Respondents were also asked to indicate their ethnic group; the mean skin tone of each group was also determined from the responses (Figure 1, Panel B). The lightest skin tones were seen in the White or Caucasian ethnic group, the darkest tones in the Black or African American ethnic group, and the other ethnic groups all had statistically similar tones in the mid-range. Since the primary goal of this study was to determine if all minority ethnic groups or people of color felt represented on the platform, the population was separated into two groups, White and POC, for additional analysis. POC consisted of participants who responded as Hispanic, Black, Asian, and “other” ethnicities.

**Figure 2:**
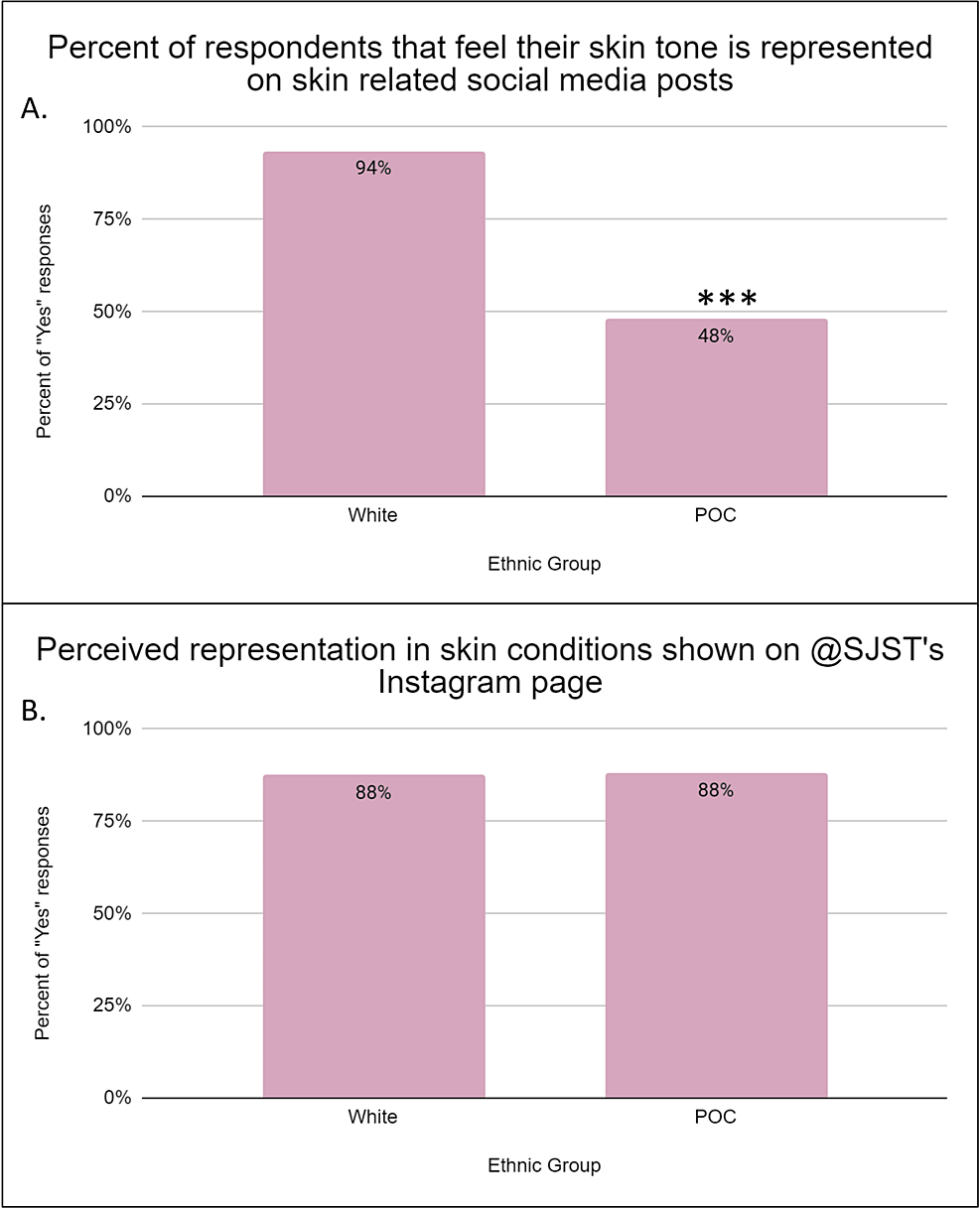
Representation of various skin tones in social media (A) This graph represents the perceived representation on social media among White and POC responders. About 88% of White individuals responded that they felt represented, while only 48% of POC said they felt represented. There was a significant difference between the two populations, with POC feeling less represented (***p < 0.00001). (B) This graph represents the perceived representation of skin conditions seen on South Jersey Skin Talk’s Instagram page. The responses were similar to this question with no statistically significant difference between the two groups. POC: People of color.

According to the results of this study, the overwhelming majority (91.5%) of respondents selected Instagram as one of their most used social media platforms, and most participants felt Instagram was "slightly useful" as an educational tool. Overall, POC was significantly less likely to feel represented by posts on social media (p < 0.00001). The majority of White respondents reported feeling represented, while less than half of POC respondents responded “yes” (Figure 2, Panel A). Remarkably, after viewing SJST, the difference between the two populations was eliminated, with 87.5% of White participants and 88% of POC participants reporting feeling represented on the page (Figure 2, Panel B).

**Figure 3:**
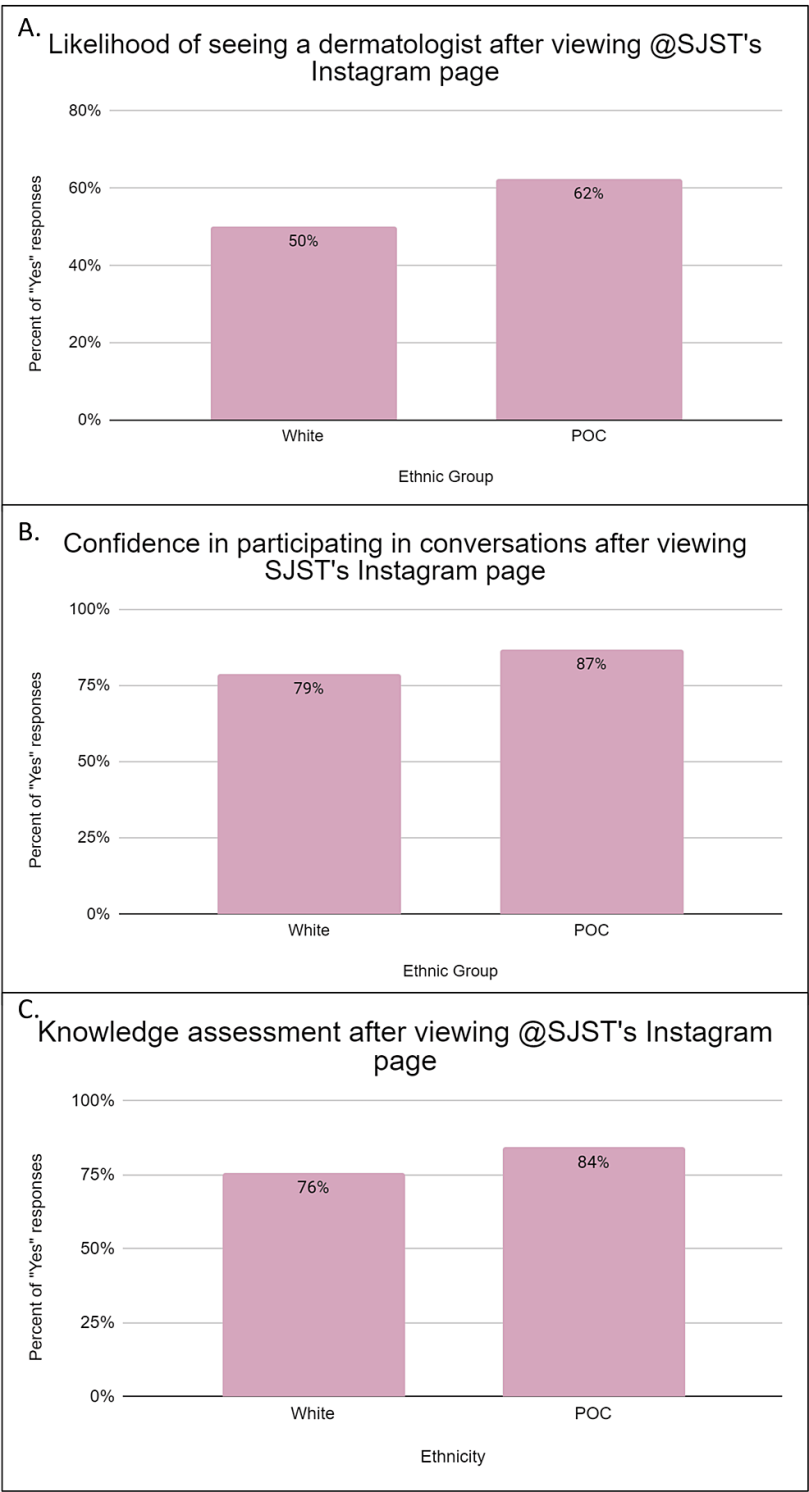
Likelihood of actively engaging in skin health discussions with confidence (A) Respondents were asked if they were more likely to see a dermatologist for their skin concerns after viewing @SJST on Instagram. Overall, 58.7% responded “yes.” POC participants responded “yes” at a higher percentage than white individuals; however, the difference was not statistically significant. (B) Participants were asked if they would feel more confident in conversations with a dermatologist after viewing @SJST on Instagram. Overall, 84.4% reported that they would have more confidence. POC participants reported slightly higher levels of confidence than White participants, but the difference was not statistically significant. (C) Participants were asked if they felt they had more knowledge about their primary skincare concern. Overall, 81.8% of responses were “Yes.” There was no statistically significant difference between these two groups. SJST: South Jersey Skin Talk; POC: People of color.

One of the major goals of this study was to empower participants, regardless of their skin tone or ethnic makeup, to participate meaningfully in their own skin health. Participants were asked if they were more likely to see a dermatologist after viewing @SJST on Instagram. According to this survey, the vast majority (92.7%) of respondents reported that SJST uses language that can be easily understood by anyone. As a result of this, more than half of the participants responded that they would be more likely to see a dermatologist, regardless of their skin tone or ethnic group (Figure 3, Panel A). The level of confidence that participants would have in having conversations with their dermatologists was also assessed as part of this study. Overall, 84.4% of participants indicated that they would have more confidence during conversations in those professional settings after viewing @SJST on Instagram (Figure 3, Panel B). Additionally, 81.8% of respondents also felt more knowledgeable about their primary dermatologic concern after viewing SJST’s posts; a breakdown by ethnicity shows that 75.80% of White respondents and 84.40% of POC respondents stated feeling more knowledgeable about their primary skin concern after viewing the page (Figure 3, Panel C). Of note is the fact that after viewing SJST, the vast majority of respondents felt that their skincare knowledge confidence was increased, with no significant difference between White and POC populations. This indicates that when various skin tones are appropriately represented, it can positively impact community health.

## Discussion

Instagram as a source of dermatologic education

Previous studies have suggested that social media serves as a potent means for facilitating social interactions and, in addition, functions as an effective tool for education and learning [21,22]. Instagram’s accessibility, visual nature, and wide-reaching user base enable it to be a powerful platform for education, especially in dermatologic content. In the realm of dermatologic education, Instagram allows dermatologists, skincare professionals, and influencers to share visually engaging and easily understandable content, such as infographics, images, and short videos directly to their audience [23,24]. Instagram’s vast audience enables dermatology experts to publicly delineate information about dermatologic health, conditions, treatments, and practices to a broad and diverse demographic [2]. By leveraging its user-friendly interface and interactive features, Instagram can foster a sense of community, encouraging individuals to learn from one another and seek professional advice for their unique dermatologic concerns, ultimately promoting better skincare practices and overall well-being.

Despite its potential benefits, Instagram also has several disadvantages as a source of dermatologic education. One of the main concerns is the lack of regulation and quality control over the information shared on the platform. While some content may come from qualified dermatologists, there is an abundance of unverified advice from skincare influencers, which can be misleading or even harmful [23-25]. Additionally, since Instagram content often lacks citations and references, users may have difficulty discerning credible information from anecdotal claims and trends. According to a study from 2018, hashtags related to dermatology evinced that out of 146 distinct Instagram influencers identified, only 5% were verified dermatologists. Similarly, an examination of the most popular dermatology posts on Instagram indicated that only 35% of unique users were healthcare workers [16].

Another disadvantage of Instagram as a source of information for dermatologic conditions is the lack of representation of conditions that affect SOC. Dermatologists themselves are inadequately represented on social media platforms in relation to content specifically addressing SOC [26]. This was confirmed by a study that showed board-certified dermatologists generated only 12% of the most popular dermatologic posts, while individuals without dermatology certification produced 88% of the top content [2]. This discrepancy poses a challenge in terms of ensuring the accuracy of information consumed by social media users. Board-certified dermatologists may help mitigate inaccuracies on social media by creating a more prominent presence on social media platforms. Another study revealed that dermatologists expressed higher concern for risk-related issues on social media than potential benefits; the leading concerns were regarding poor patient care, nonevidence-based information, and breaching patient privacy. However, millennials and baby boomers showed more optimism and enthusiasm toward social media use compared to Generation X dermatologists, and a significant majority (82.4%) of dermatologists planned on increasing their social media usage in the future [27]. By creating a greater social media presence, dermatologists can post inclusive, evidenced-based educational resources for all skin tones. Medical students also have a distinct advantage in becoming agents of change due to their access to up-to-date medical knowledge and their ability to communicate complex information in an easily understandable manner. Their position as emerging healthcare professionals allows them to bridge the gap between medical expertise and public awareness, fostering inclusive and accurate discussions about skin health for diverse populations.

South Jersey Skin Talk

SJST is an organization created by medical students from the RowanSOM. The mission of this community outreach initiative is to increase health literacy in SOC, particularly in underserved communities. SJST places an emphasis on depicting dermatologic conditions on a variety of skin colors to provide a more inclusive representation of skin conditions that affect POC on social media. SJST uses reputable resources, such as the American Academy of Dermatology, along with information from RowanSOM’s medical school dermatology curriculum. The goals of SJST include addressing dermatologic health disparities, empowering patient autonomy, increasing access to healthcare and resources, and increasing representation and inclusivity.

Representation is extremely important in healthcare. According to this study, POC felt less represented on social media compared to White respondents prior to visiting SJST on Instagram (p < 0.00001). However, all respondents felt equally represented when viewing SJST’s posts (Figure 2). This data confirms the initial hypothesis by demonstrating that community interventions targeted at skin health for POC play a crucial role in addressing the underrepresentation of diverse skin tones in social media, medical literature, research, and clinical practices. As such, SJST helps highlight the experiences and concerns of individuals with different skin colors. In a study exploring the perception of Black patients and their dermatologic care, participants reported that dermatologists with expertise in treating black skin and hair delivered particularly valuable care to black patients. These findings imply that enhancing dermatologists’ training in SOC, cultural competency, and empathic communication, along with promoting greater diversity in the dermatology workforce, could significantly increase Black patients’ satisfaction with dermatologic care [28]. The first step toward achieving this goal is creating more representation for POC on social media and in medical education.

POC often face unique challenges related to skin health and may be more prone to certain skin conditions, such as hyperpigmentation, keloids, and eczema, and certain types of skin cancer, such as acral lentiginous melanoma [5]. SJST helps bridge the gap in healthcare disparities by providing targeted education, resources, and support to address these specific concerns. SJST’s commitment to diversity in dermatology is exemplified in the number of images showcasing conditions in SOC versus light skin. About 90.4% of posts showcase SOC images and 82.7% showcase images with lighter skin. The nearly equal representation on the page is mirrored by the results, where 87.5% and 87.8% of White and POC respondents, respectively, reported feeling represented on SJST’s Instagram (Figure 2, Panel B). Community interventions focused on skin health for POC such as SJST empower individuals by providing them with knowledge and tools to take better care of their skin. In fact, based on this survey, 86.80% of POC respondents report that they would feel more confident participating in conversations with their dermatologist regarding their primary dermatologic concern (Figure 3, Panel B). Greater confidence in discussing health concerns increases patient autonomy, which cultivates knowledgeable individuals who can partake in making informed decisions about their healthcare plans. This, in turn, leads to better treatment adherence, improved health outcomes, and stronger patient-physician relationships.

Many POC may face barriers to accessing dermatologic healthcare services. SJST is based in Camden County, New Jersey, which is one of the most vulnerable areas in NJ [29]. According to a survey done by the New Jersey State Policy Lab, Black and Hispanic residents faced greater challenges in accessing high-quality health care compared to their White counterparts. In New Jersey, 26% of Black residents and 26% of Hispanic residents reported poor access, while only 12% of White residents reported the same. These statistics unmistakably illustrate that POC in New Jersey encounter more obstacles when trying to access healthcare services [30]. SJST strives to help overcome these barriers by providing information about affordable or free healthcare options and community resources. By connecting individuals with the right resources in easily understandable verbiage, community interventions such as SJST can ensure that POC have access to adequate care and support for their skin health needs.

Limitations

There are several limitations of this study, the first of which includes the scope of the survey. This survey was conducted solely through social media platforms, which may not fully represent the diverse population, especially individuals who do not use these social media platforms or have limited access to the internet. However, the survey was distributed in many different social media groups to reach a sample size representative of the general population. Additionally, data was not collected on how many responses came from participants who found the survey on Instagram. The study also only collected data during an eight-week period, which may not capture the long-term impact of SJST’s intervention on health literacy or representation. The data collected through the survey relied on self-reported responses, which may be subject to bias. There was also a varied number of participants that responded to each question, which can further reduce the reliability and generalizability of the survey. The survey was only created in English, which would exclude participants who do not read in English.

## Conclusions

Community interventions focusing on skin health for POC are an integral part of addressing healthcare disparities, promoting inclusivity, empowering individuals, and ensuring equitable access to quality dermatological care. These interventions play a vital role in improving the overall health and well-being of POC communities. This survey study evaluated the impact of SJST (@sjskintalk), a medical student-led organization on Instagram, in enhancing health literacy in individuals with SOC. After viewing SJST’s page, both White and POC participants reported feeling more represented, and a significant percentage of respondents from both groups felt more knowledgeable about their primary skin concerns. Further research should be conducted to investigate the impact of other health disparities and healthcare outcomes for SOC. This study aimed to inspire others to implement more dermatologic community outreach initiatives targeted at educating POC communities.
